# Monitoring real-time hormone release kinetics *via* high-content 3-D imaging of compensatory endocytosis†
†Electronic supplementary information (ESI) available. See DOI: 10.1039/c8lc00417j


**DOI:** 10.1039/c8lc00417j

**Published:** 2018-07-30

**Authors:** Andrei I. Tarasov, Juris Galvanovskis, Olof Rorsman, Alexander Hamilton, Elisa Vergari, Paul R. V. Johnson, Frank Reimann, Frances M. Ashcroft, Patrik Rorsman

**Affiliations:** a Oxford Centre for Diabetes, Endocrinology and Metabolism , Churchill Hospital , University of Oxford , Headington , OX3 7LE , Oxford , UK . Email: andrei.tarasov@ocdem.ox.ac.uk ; Email: patrik.rorsman@hmc.ox.ac.uk; b Oxford National Institute for Health Research , Biomedical Research Centre , Churchill Hospital , Oxford OX3 7LE , UK; c Metabolic Research Laboratories , Wellcome Trust–MRC Institute of Metabolic Science , Addenbrooke's Hospital , Cambridge , CB2 0QQ UK; d Department of Physiology, Anatomy and Genetics , University of Oxford , Parks road , Oxford , OX1 3PT , UK; e Institute of Neuroscience of Physiology , Department of Physiology , Metabolic Research Unit , University of Göteborg , Box 430 , SE-405 30 Göteborg , Sweden

## Abstract

High-content real-time imaging of hormone secretion in tissues or cell populations is a challenging task, which is unlikely to be resolved directly, despite immense translational value. We approach this problem indirectly, using compensatory endocytosis, a process that closely follows exocytosis in the cell, as a surrogate read-out for secretion. The tissue is immobilized in an open-air perifusion chamber and imaged using a two-photon microscope. A fluorescent polar tracer, perifused through the experimental circuit, gets trapped into the cells *via* endocytosis, and is quantified using a feature-detection algorithm. The signal of the tracer that accumulates into the endocytotic system reliably reflects stimulated exocytosis, which is demonstrated *via* co-imaging of the latter using existing reporters. A high signal-to-noise ratio and compatibility with multisensor imaging affords the real-time quantification of the secretion at the tissue/population level, whereas the cumulative nature of the signal allows imprinting of the “secretory history” within each cell. The technology works for several cell types, reflects disease progression and can be used for human tissue.

## Abstract 2

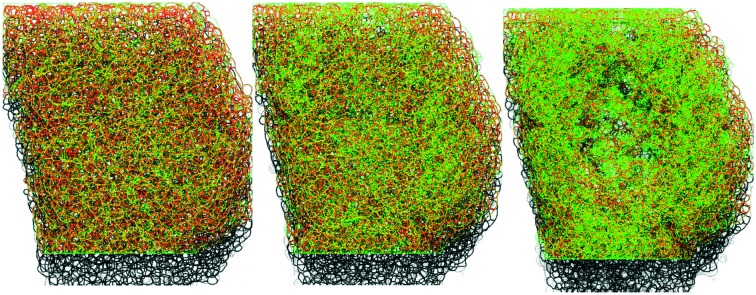
A novel technology for quantifying hormone secretion from tissues, with a single-cell resolution.

## Introduction

Cell-to-cell heterogeneity in hormone secretion can provide key information as for the nature and progression of endocrine disorders such as diabetes mellitus.^1^ Despite its potentially high clinical relevance, modern technology for real-time single-cell imaging of secretion cannot rival established macroscopic techniques based on radioimmunoassay (RIA) or enzyme-linked immunosorbent assay (ELISA). Microscopic techniques for visualization of exocytosis conventionally rely on the detection of single-vesicle fusion events, as fast (<1 s) and transient increases in fluorescence intensity. In practice, however, single-vesicle imaging techniques, such as the total internal reflection fluorescence (TIRF) microscopy of fluorescent cargo^2^ or the two-photon detection of a polar tracer,^3^ are limited to small groups of cells (Movie S1†). In addition, the cells are subject to photodamage, due to fast imaging rates, which limit the duration of the experiment.

**Fig. 1:**
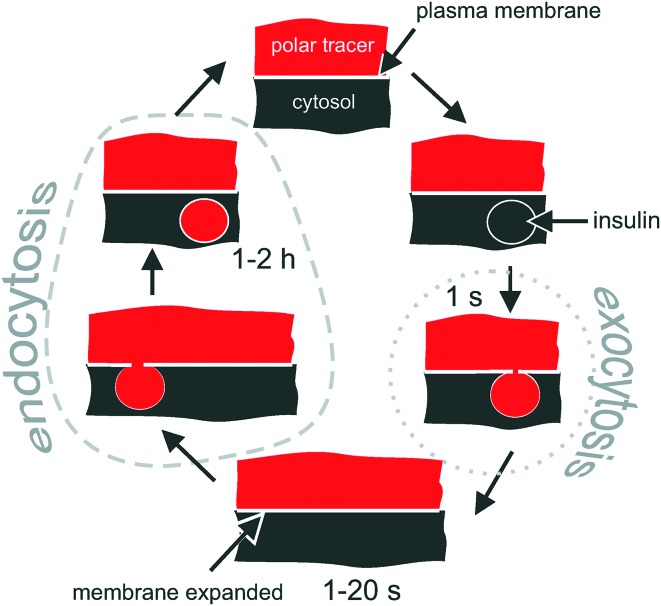
Intracellular fate of a polar tracer during the exocytosis–endocytosis coupling in excitable cells. Conventional exocytosis starts with docking of the vesicle, followed by formation of a transient (1 s) pore between the vesicle and the extracellular solution, which gets immediately filled with the polar tracer. The vesicle then collapses into the plasma membrane. When monitored by 2-photon microscopy, the process appears as a bright short-living (1 s) maximum. Due to the fusion of the intracellular vesicle, the plasma membrane expands, which is followed (in 1–20 s) by the retraction of the membrane (endocytosis). During the retraction, some extracellular polar tracer is trapped inside the newly formed vesicle. The vesicle is detectable for 1–2 hours *via* 2-photon microscopy when SRB (MW = 0.56 kDa) is used as a polar tracer.

Single-vesicle fusion *per se* has little translational value, as hormone secretion deficiencies mostly stem from defects in metabolism and signalling. At the same time, the physiological processes linked to hormone secretion (in the case of insulin, such as changes in β-cell energy metabolites) operate on the timescale of minutes/tens of minutes.^4^ The typical secretory response of an isolated pancreatic islet of Langerhans extends for over an hour,^5^ which agrees well with the timescale of correction of blood glucose levels in humans.^6^ Therefore, whilst recording insulin secretion at a one second interval seems quite logical – as this is its natural temporal domain – the translational value of a long recording, not necessarily with a high sampling rate, is much higher. This suggests an alternative avenue: moving away from single-vesicle detection and looking for a technologically realistic surrogate for the quantification of exocytosis. A notable progress was achieved in imaging pancreatic islets of Langerhans, the organoids that produce the principal hormones regulating blood glucose levels, insulin and glucagon. The insulin crystal is naturally stabilized by two zinc ions, which are then co-released with the hormone by the pancreatic islet β-cell.^7^ The kinetics of insulin secretion was therefore quantified by monitoring the release of Zn^2+^ from the islets using soluble^8^ or membrane-bound^9^ probes. We approached the problem from a different angle and used fluid-phase compensatory endocytosis as a surrogate reporter for secretion. In neurons and endocrine cells, endocytosis is a form of post-secretory membrane retrieval, which is tightly spatiotemporally coupled to exocytosis^10^,^11^ (Fig. 1). The mechanism of the coupling is yet unclear; the likely candidates include membrane tension,^12^ synaptotagmin I,^13^ SNARE proteins,^14^ and Ca^2+^/calmodulin.^15^ The spatial coupling between exocytosis and endocytosis is not absolutely precise:^16^ the two processes are likely to occur close to each other but do not necessarily use the same vesicle. The temporal association between the two signals is quite strong, with a delay between the two processes ranging from milliseconds to seconds in neurons^16^ and seconds to tens of seconds in pancreatic β-cells.^17^,^18^

## Experimental

### Animals, tissue isolation and culture

NMRI mice (Charles River, Harlow, UK) were used throughout the study, apart from the experiments involving genetic manipulation of the K_ATP_ channel and fumarate hydratase (Fh). Fh^–/–^, Ins-ChR2, Glu-Venus and Sst-Cre/Rosa26tdRFP/GCaMP3 (“Sst-RFP”) mice were developed as described.^19^–^22^ Age- and sex-matched wild-type animals were used as controls. The mice were kept in a conventional vivarium with a 12 hour-dark/12 hour-light cycle and *ad libitum* access to food and water and were killed by cervical dislocation. All mouse experiments were conducted in accordance with the United Kingdom Animals (Scientific Procedures) Act (1986) and the University of Oxford ethical guidelines.

Pancreatic islets were isolated from the mice by injecting collagenase solution into the bile duct, with subsequent digestion of the connective and exocrine pancreatic tissue. The islets were picked up using a P20 pipette, under a dissection microscope and cultured overnight in RPMI medium containing 11 mM glucose, supplemented with 10% FBS, 100 IU mL^–1^ penicillin and 100 μg mL^–1^ streptomycin (all reagents were obtained from Life Technologies, Paisley, UK).

Human pancreatic islets were isolated in the Oxford Diabetes Research & Wellness Foundation Human Islet Isolation Facility according to published protocols.^23^,^24^ Informed signed consents were obtained from the donors. The islets were prepared for imaging as described above for their mouse counterparts.

### Microperifusion chamber

**Fig. 2:**
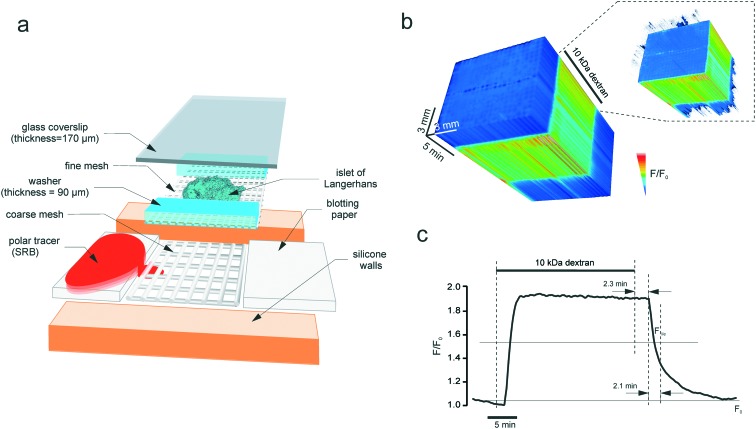
The microperifusion (microblotting) imaging chamber. (a) The assembly of the chamber. The basin has a plastic base, with silicone walls attached by suction. Perifusion is done through a 10 mm canal by using blotting paper: the inflow and the outflow are collected at the same rate. The islet is positioned into a scaffold formed from a coarse (thickness = 200 μm, unit size = 200 μm) and a fine (thickness = 200 μm, unit size = 200 μm) mesh. The coarse mesh supports the structure whereas the fine mesh immobilises the specimen (islet) itself. The islet rests between the 90 μm-thick cellophane walls that limit the degree of deformation that it receives upon sealing with the glass coverslip. Polar tracer SRB is blotted through the sandwich scaffold containing the imaged object, islet of Langerhans. (b) and (c) The kinetics of the fluorescence of 10 kDa dextran (0.1 mM) perifused through the chamber at 50 μL min^–1^. (b) The timelapse image of the chamber acquired every 5 s as a 3D stack. The intensity was normalised to the first frame, pixel-by-pixel, and visualised according to the look-up table. Inset: The thresholded 3D stack showing the onset of the dextran fluorescence along the chamber. (c) Line plot of the fluorescence kinetics. At this perifusion speed, the circuit has a lag time of 2.3 minutes and a characteristic clearance rate of 2.1 minutes.

A round silicone washer (thickness = 375 μM, Harvard apparatus) was secured to a dust-free plastic base (*e.g.* 75 mm petri dish, Sarstedt). An opening was cut in the washer, to create a basin on the plastic base. A piece of thick nylon mesh (unit size = 160 μM, thickness = 200 μM, Harvard apparatus) was placed into the chamber, and a fine nylon mesh (unit size = 46 μM^25^) supplemented with 90 μM plastic washers was positioned on top of the thick mesh (Fig. 2a). The inflow and outflow pieces were manufactured using small pieces of blotting paper, the outflow piece being used as a small roll. This is needed to ensure that the outflow piece catches the solution that drips off the roll in a gravity-dependent manner. Together with the inflow paper, the outflow paper provided hydrodynamic isolation from any possible perturbations of flow in the perifusion system (induced by a peristaltic pump, air bubbles, *etc.*). The flow inside the chamber relies on the capillary effect. The solution was introduced into the system and the tissue (pancreatic islet) was placed on the fine mesh, straight between the washers. A 26 × 40 mm coverslip (thickness = 0.17 mm) was secured on top of the silicone washer. The coverslip very gently pressed the tissue thereby immobilizing it inside the chamber, the washer serving as a wall limiting the tissue deformation. The chamber was connected to the perifusion system (Ismatec REGLO MS-2/12 peristaltic pump, capillaries, 19G needles) that provided the continuous flow at 50 μL min^–1^ and positioned inside the heated stage (+34 °C), under the upright microscope.

### Imaging of endocytosis, [Ca^2+^]_i_, [ATP/ADP]_i_, [Zn^2+^]_e_ and targeted sensors in the islet cells

Time-lapse 2-D and 3-D imaging was performed on a LSM510-Meta scanning confocal system based on an Axioskop-FS2 microscope, using a C-Apochromat 40×/1.2 water objective (Carl Zeiss). The islets immobilized in the microchamber (as above) were perifused with EC solution containing (mM): 140 NaCl, 4.6 KCl, 2.6 CaCl_2_, 1.2 MgCl_2_, 1 NaH_2_PO_4_, 5 NaHCO_3_, 10 HEPES, (pH 7.4, with NaOH), 1 sulforhodamine B (SRB) and 0.2% BSA. SRB was excited at 900 nm (2-photon mode) using a Chameleon Vision-S femtosecond laser (Coherent) with the GDD set to minimum, and 2D images were acquired at 550–600 nm using the diffraction grid (S channel). FITC (Fig. 4a) was excited at 940 nm using the same laser. [Ca^2+^]_i_ [ATP]_i_, and [Zn^2+^]_e_ were reported using Fluo4 (Molecular Probes), Perceval^26^,^27^ and Zimir,^9^ respectively. Fluo4 and Zimir were pre-loaded into the islet cells/cell membrane by a 60–90 min pre-incubation in 6 μM of each dye. Perceval (a gift from Prof Gary Yellen, Harvard University, Boston, U.S.A.) and NPY-Venus (a gift from Prof Atsushi Miyawaki, RIKEN BSI, Wako, Japan) were delivered adenovirally, with a 24–36 h expression time. All the green fluorophores apart from FITC were excited at 488 nm (single-photon mode) and the emission was collected between 510 and 540 nm. RFP (Fig. 6a) was excited at 543 nm and the emission was collected between 570 and 630 nm. ChR2 (Fig. 4e) was excited at 470 nm. The acquisition was performed at 512 × 512 using the frame scanning mode with a pixel dwell time of 6 μs, at an interval of 22–36 s using ZenBlack software (Carl Zeiss). For the experiments on FITC-dextran (Fig. 4a) and the human islets (Fig. 6b), the 1024 × 1024 mode was used.

### Single-islet hormone release measurements

**Fig. 3:**
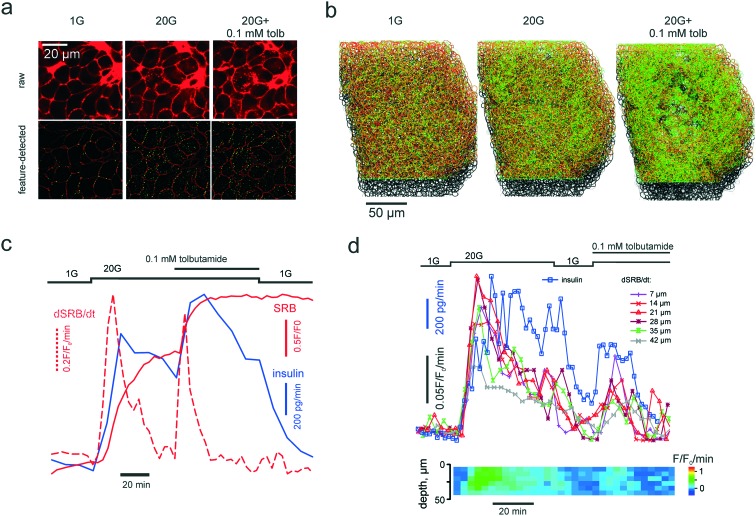
PAPT reflects the macroscopic secretory kinetics. (a) top: Raw unprocessed images of cells within the islet of Langerhans, exposed to extracellular solution containing 1 mM glucose and 20 mM glucose in the absence and presence of tolbutamide, as indicated. (a) bottom: Images after feature detection, presenting the extracellular space (“cell borders”) (red) and PAPT maxima (green). (b) Rendering of 3-D images of the whole islet after the feature detection, color-coded as in (a) bottom. (c) and (d) Kinetics of the endocytosis imaged in a single islet simultaneously with quantification of total insulin secretion (using RIA) showing the global figures (c) and the in-depth contribution of tissue layers and phasic changes in insulin secretion (d).

Single islets were immobilized inside the microchamber under the microscope and perifused with the EC solution lacking SRB and containing 1 mM glucose and 100 μM 3-isobutyl-1-methylxanthine for >30 min. After SRB was introduced into the system, 2D or 3D imaging data acquisition was initiated and the collection of the flow-through fractions was started every 5 (Fig. 3c) or every 2 (Fig. 3d) minutes. The insulin contents were determined as described elsewhere^28^ using a RIA from Eurodiagnostica (p/n RH310; Malmo, Sweden).

### Data analysis

**Fig. 4:**
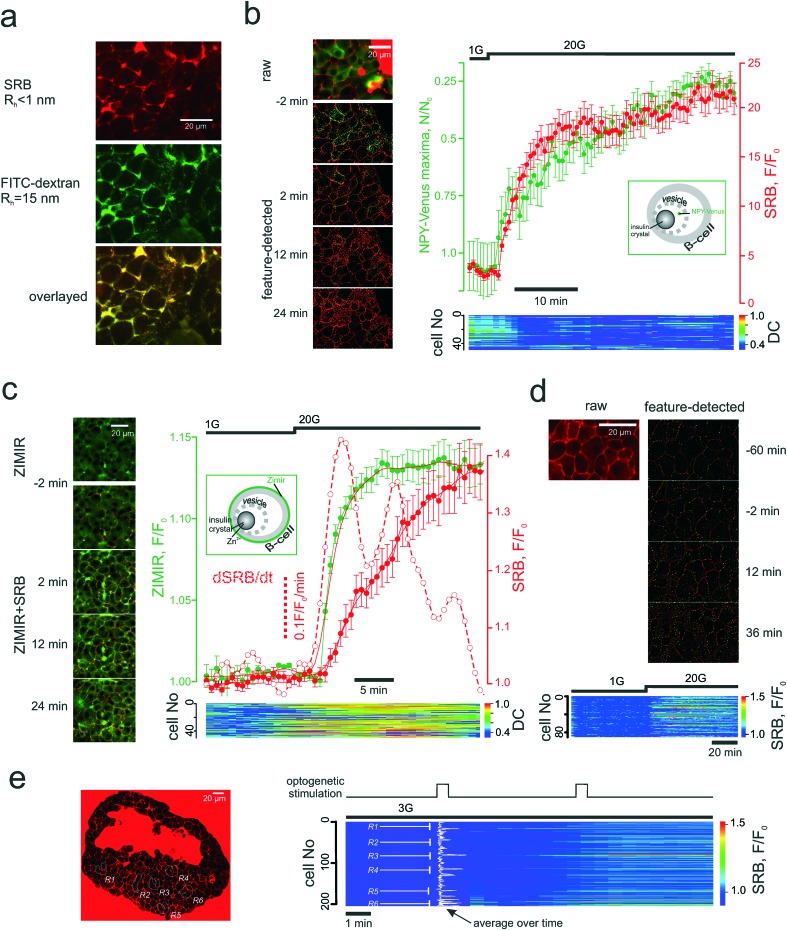
PAPT closely follows the microscopic secretory kinetics. (a) Co-imaging of SRB (top) and fluorescently tagged dextran (middle) and co-localization of the signals inside the islet cells (bottom). (b) and (c) Co-imaging of SRB endocytosis and loss of NPY-Venus (b) or increase in Zimir fluorescence (c) and the kinetics of the two experiments. Time course images (left) of the signals before and after the stimulation by glucose and representative mean traces (right) for the *n* = 6 islets (NPY) and *n* = 7 islets (Zimir) experiments. Bottom: Heat-map plots representing the distance correlation (“DC”) between the NPY-Venus and SRB (b) or Zimir and SRB (c) signals in individual cells. Insets: Schematic showing the targeting of the two exocytotic reporters; (d) SRB endocytosis upon long-term exposure without stimulation of exocytosis. Top: The features detected (endocytotic maxima) are given in green. Bottom: Time course of the SRB endocytosis within the islet cell population. Glucose was elevated after 1 h and produced a prompt stimulation of the SRN uptake; (e) light-induced SRB endocytosis in islets isolated from Ins-Chr2 mice. The induction was performed at the regions R1-R6 (left), as indicated in the populational time course (right).

Image sequences were analysed (feature detection, segmentation and ROI intensity *vs.* time analysis) using a purposely-developed Java plug-in for open-source FIJI software (; http://fiji.sc/Fiji) deposited at ref. 29. In brief, the maxima are detected *via* an adjustable difference of Gaussians (DoG), which is followed by DoG-based tissue segmentation into individual cells. The maxima associated with cell membrane and extracellular tracer are discarded by analysing the Hessian matrix. The Hessian matrix routine also allows discarding the maxima associated with the bulk mode endocytosis. The two forms of compensatory endocytosis that we believe to be involved in our system, (i) clathrin-mediated and (ii) bulk modes, can theoretically be differentiated by the appearance of the endocytotic pattern (dotted maxima for (i) and bulky uptake for (ii)) and the onset time (seconds for (i) and minutes for (ii)).^10^ It is the delayed kinetics of the bulk mode that made us rule it out of the consideration. At the same time, when quantifying the PAPT effect, we found it more useful to account for the total increase of the fluorescence intensity inside the patterned system rather than just the increase in numbers of the maxima. This accounts for the network nature of the endocytotic/endosomal system^30^ (which can be seen *e.g.* in Fig. 4a).

The numerical data was analysed using IgorPro (Wavemetrics) and Matlab (Mathworks). The experiments on the human islets were performed on the islets isolated from the *n* = 3 donors. Statistical analysis was performed using R.^31^ Data is presented as the mean values ± S.E.M. Comparisons within one experiment were performed using the Kruskall–Wallis test with Nemenyi post-hoc analysis (independent samples) or the Friedman test with Nemenyi post-hoc analysis (dependent samples). Differences with *p* < 0.05 were considered statistically significant.

## Results and discussion

### Perifusion chamber

The conventional method for recording the dynamics of insulin vesicle exocytosis involves perifusion of the islet with the extracellular polar tracer SRB (sulforhodamine B)^32^–^34^ and two-photon imaging of the fusion events (Movie S1†). The endocytotic maxima are of a similar dimension (<200 nm) hence their real-time detection within the turbid medium requires a sensitive objective (of a high numerical aperture and a low working distance) and a mechanically stable microperifusion circuit, which is especially critical for long time-lapse imaging experiments. This requirement limits the potential use of microfluidics approaches, developed for islet multimodal imaging^35^,^36^ in the present study. We opted for an upright system with physiologically relevant^37^ rates of perifusion flow and safeguarded it against motion artefacts (Fig. 2a). The isolated pancreatic islets were positioned inside a sandwich nylon scaffold and gently pressed by the glass coverslip (Fig. 2a). Not only did this allow for the immobilization of the tissue but it also permitted the mechanical alignment of multiple cells from the islet outer layer within the same optical plane. This alignment is critical for the 2D imaging of multiple cells using the confocal microscope, as the trappable dyes or virally delivered constructs are predominantly loaded/expressed in the cells located in the outer layer of the tissue. To attenuate any movements induced by the perifusion system or air bubbles, we supplemented the sandwich imaging configuration with a paper feeder and outflow (Fig. 2a) and thus blotted (rather than perifused) the solution through the chamber. Imaging the chamber flow with 10 kDa dextran conjugated with a fluorophore tetramethylrhodamine reported fast and uniform onset and offset of the fluorescence over time (Fig. 2b). At the flow rate of 50 μL min^–1^, the circuit has a 2.3 min lag period and a characteristic clearance time of 2.1 min (Fig. 2c).

### Patterned uptake *vs.* bulk insulin secretion

Once introduced into the perifusion system, the polar tracer enters all the extracellular space (Fig. 3a), thereby sharply outlining the individual cells within the islet. We generally considered cells with a high and uniform uptake of SRB as non-viable, however, in freshly isolated islets there was a population of “morphologically viable” cells that took up the tracer quite intensely (Fig. S1a†), echoing previous reports in astroglia.^38^ These cells were classified as vascular endothelium by positive lectin staining (Fig. S1a†). The uptake of SRB by lectin-negative pancreatic islet cells is otherwise very low at basal glucose (1 mM) (Fig. 3a) and, if present, bears a patchy pattern, detectable with the DoG-based algorithm^29^ (Fig. 3a and b). An increase in the extracellular glucose or addition of the K_ATP_ channel blocker tolbutamide to the perifusion medium stimulates exocytosis and endocytosis, seen as dramatic increases in the number of the fluorescent maxima inside the islet cells (Fig. 3a and b, Movie 2 and 3†). Measured in single islets, the macroscopic kinetics of this p[combining low line]atterned a[combining low line]ccumulation of the p[combining low line]olar t[combining low line]racer (PAPT) correlated very closely with the whole-islet insulin secretion as detected by the radioimmunoassay (Fig. 3c). Importantly, the peaks in the first temporal derivative of the PAPT closely coincided with the stimulus-induced maxima in insulin secretion. However, the relaxation to the basal values was much slower for the soluble insulin signal (Fig. 3c), with the characteristic times reaching up to 16 minutes (*cf.* with 2.1 minute for the perifusion flow alone, Fig. 2b). This *ca.* 10 minute delay may look at odds with the amperometry result,^39^ reporting the fast relaxation of currents induced by insulin or serotonin release from single β-cells. Amperometry reports the soluble insulin in the cell vicinity, although the peptide is stored by the β-cell in the crystalline form.^40^ Intravesicular solubilisation of insulin would substantially increase the osmotic pressure,^41^ hence the hormone is expected to be secreted in its crystalline form. We therefore believe that the delay reflects the time needed for the dissolution of insulin crystals (>7 min in blood plasma^42^).

PAPT was not limited to the outer layer of the islet: cells situated as deep as 50 μm from the surface also displayed strong responses to glucose (Fig. 3d). A collection of the secreted hormone at a higher frequency allowed resolving phasic changes at this signal with 20 mM glucose, which, again, closely correlated with the temporal derivative of the PAPT signal (dPAPT/d*t*; Fig. 3d).

Pancreatic β-cells express α_2_ adrenergic receptors as well as receptors to somatostatin (SSTRs), a hormone secreted by neighbouring δ-cells. Both adrenaline and somatostatin are known to attenuate glucose-induced insulin secretion by membrane repolarization and a direct inhibitory effect on insulin exocytosis.^43^ Application of the α_2_ agonist clonidine arrested PAPT whilst the α_2_ and SSTR antagonists yohimbine and CYN154806, respectively, stimulated the uptake (Fig. S1b and c†)

### Patterned uptake *vs.* other single-cell secretion reporters

The endocytotic nature of PAPT was confirmed by co-imaging the glucose-induced uptake with the polar tracers of different m[combining low line]olecular w[combining low line]eight (MW). The pattern of the tracer uptake, which appeared as a structured network rather than a set of maxima, at a higher resolution, looked very similar when outlined with SRB (MW = 0.56 kDa, hydrodynamic radius, *R*_h_ = 0.7nm (ref. 32)) or FITC-conjugated dextran (MW = 0.5 MDa, *R*_h_ > 15nm (ref. 44)) (Fig. 4a). One of the three classified^10^ modes of compensatory endocytosis, the kiss-and-run mode, features the formation of a short-living pore that mediates the mass-exchange between the secretory vesicle and the extracellular milieu. This is a special case when exocytosis and endocytosis utilize the same vesicle, which avoids full fusion. The kiss-and run pore has a maximal reported radius of ≤0.7–0.8 nm,^17^,^45^ much smaller than the linear sizes of the dextran molecule (above) or hexameric insulin (*R*_h_ = 5.6 nm (ref. 46 and 47)). SRB has been shown to track kiss-and-run exo/endocytosis in the chromaffin cell line PC12,^45^ therefore, given the data (Fig. 4a), we believe that PAPT is likely to reflect predominantly two other endocytotic modes, clathrin-mediated endocytosis and bulk endocytosis (also regarded as activity-dependent bulk endocytosis, ADBE^48^). Both modes are known to follow the exocytotic event at an interval of seconds.^10^

We then probed the temporal association of the endocytotic and exocytotic signals in the islet cells. NPY-Venus co-localizes with insulin in the β-cell secretory vesicles and is co-released with the hormone.^49^ In this work, the intracellular patterned NPY-Venus signal decreased during glucose stimulation, which correlated with an increased PAPT signal (Fig. 4b). We observed no co-localization of the two signals. Another surrogate marker of insulin secretion is Zn^2+^, which is naturally co-released with insulin.^7^ The Zn^2+^ release, reported by the extracellular membrane-bound sensor Zimir,^9^ displayed a fast monophasic jump in response to increased extracellular glucose after a delay of 2 min (corresponding to metabolic degradation of glucose) (Fig. 4c). Overall, the Zn^2+^ release kinetics was faster than that of PAPT (Fig. S1d) but correlated well with dPAPT/d*t*. The discrepancy between the PAPT and Zn^2+^ kinetics can be attributed to the either the difference in R_h_ of insulin and Zn^2+^ or the more rapid solvation of the Zn-insulin crystal following chelation of Zn^2+^ by the indicator.^50^

### PAPT reflects stimulated endocytosis in a heterogenous population

Theoretically, the application of the PAPT technology may be limited by constitutive endocytotic signals that occur in the absence of the stimulation. We therefore measured the rate of the non-stimulated PAPT and found it comparatively low: even after an hour-long perifusion at basal glucose the accumulation of SRB was small (Fig. 4d). Likewise, PAPT stimulated by 20 mM glucose in the presence of a K_ATP_ channel opener diazoxide was small but progressed significantly upon removal of the drug (Fig. S1e†).

Another aspect defining the range of potential applications is the ability to adequately report the signal from a heterogeneous cellular population. We probed this using the Ins-ChR2 mice in which the depolarization of β-cells can be precisely timed and localized using light-induced activation of depolarizing membrane currents. In the islets from these mice, the stimulation can be restricted to a particular group of cells. When applied at six different regions throughout the islet syncytium at substimulatory 3 mM glucose, the first 30s light pulse triggered an immediate response in only one of the regions (R6 in Fig. 4e) and induced a slower onset in the other regions (R1 and R3). However, the second light pulse prompted an immediate and nearly saturating response within and even outside the illuminated regions (Fig. 4e and S1f†). We believe the reason for that is the tight electrical coupling between the neighbour cells inside the islets, which may allow the propagation of the excitatory depolarizing stimulus, even at low glucose concetrations.^51^ Nevertheless, the PAPT from the stimulated regions remained much stronger than that from the non-stimulated regions reflecting a clear heterogeneity of the stimulus/response.

### Parallel imaging: secretion correlates with metabolic activity

**Fig. 5:**
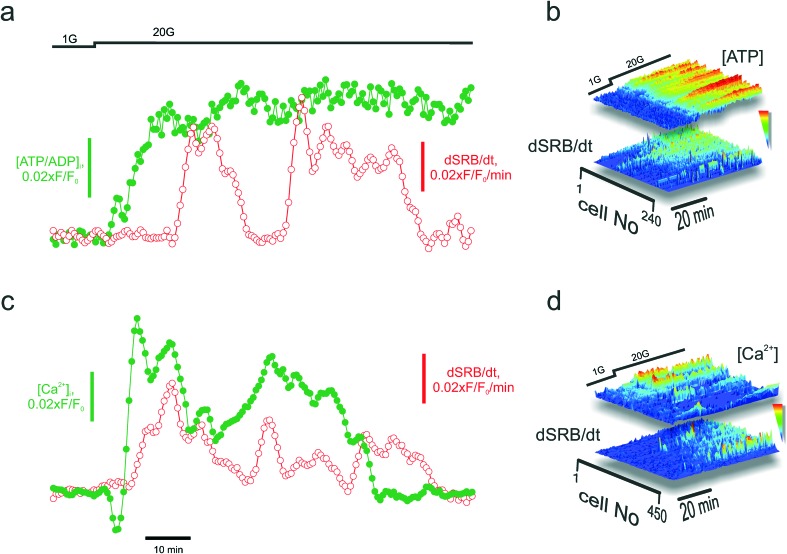
Applications: multiparameter imaging. Parallel imaging of secretion (*via* SRB endocytosis) and metabolism (cytosolic ATP/ADP, using Perceval) (a) and (b) or signalling (Ca^2+^, using Fluo4) (c) and (d) in the islets, with a single-cell resolution. (a) and (c): Average secretion (dSRB/d*t*) and ATP/ADP (a) or [Ca^2+^]_i_ (c) per islet. (b) and (d): Surface plots reporting the kinetics of endocytosis in individual cells (dSRB/d*t*) and ATP (b) or [Ca^2+^]_i_ (d) in the same cells within the islets. The parallel signals have been sorted according to the magnitude of the ATP/ADP (b) or [Ca^2+^]_i_ (d) response over a *ca.* 40 minute period following the stimulation with high glucose concentrations. Note that the best “PAPTers” are not the cells with the most prominent Ca^2+^ dynamics.

One of the applications for the high-content technology for imaging secretion is the opportunity it affords to perform parallel measurements of upstream signals and assess the sensitivity to those on the cellular level. Insulin secretion is believed to be triggered by the metabolically-driven elevation of cytosolic Ca^2+^ ([Ca^2+^]_i_) but amplified by additional intracellular signals such as ATP, cAMP, and glutamate.^52^ We tested the feasibility of monitoring PAPT in parallel with measuring the intracellular ATP/ADP ratio (using the reporter Perceval^27^) or [Ca^2+^]_i_ (using the indicator Fluo4) (Fig. S1g†). An increase in the extracellular glucose concentration from 1 to 20 mM induced the increase in the ATP/ADP ratio (Fig. 5a) and [Ca^2+^]_i_ (Fig. 5a), which, in turn, was followed by PAPT (Fig. 5a and c, respectively). The whole-islet data reflected strong causal links between the increase in cell metabolism and Ca^2+^ dynamics and PAPT, on a per-islet basis. Analysis of the parallel data over a physiologically relevant time interval (tens of minutes for both *in vivo*^6^ and in the isolated islets^5^), on a per-cell basis, provided another valuable insight. The per-cell correlation between PAPT and the metabolic state was strong (Fig. 5b). However, it was lower in the case of [Ca^2+^]_i_ and PAPT (Fig. 5d). Tentatively, this suggests that although [Ca^2+^]_i_ is required to initiate exocytosis (Fig. S2†), it may be less important at a later progression of the secretory response known as the amplifying stage,^52^ at which other factors of the metabolic nature^4^,^53^–^56^ have been suggested to modulate insulin secretion.

### PAPT works in other cell types and species

**Fig. 6:**
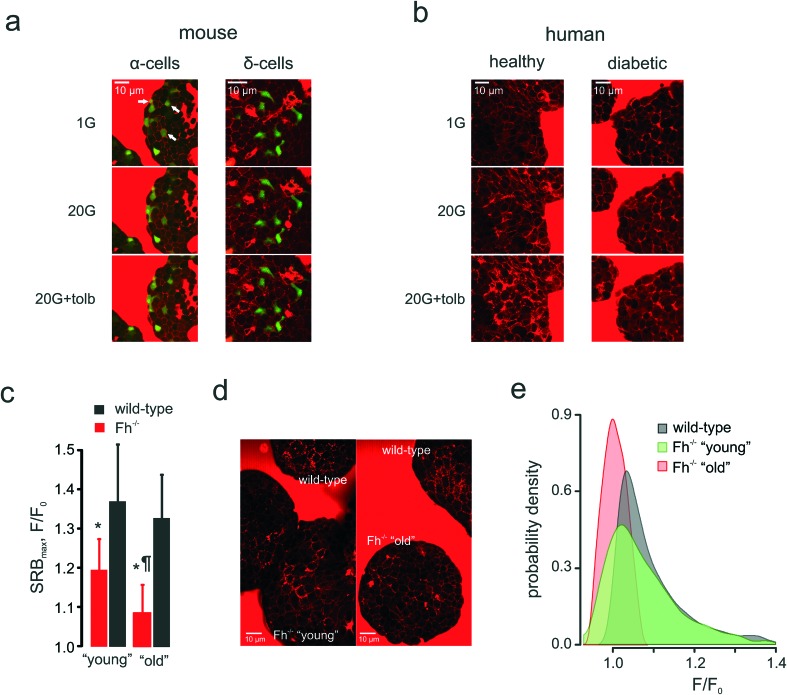
Applications: cell heterogeneity and clinical impact. (a) and (b): Glucose-induced compensatory endocytosis at 1 mM and 20 mM glucose in mouse islet α-cells (representative of *n* = 15 islets) and δ-cells (representative of *n* = 11 islets) (a) and human islets from healthy (representative of *n* = 15 islets from 3 donors) and type 2 diabetic (representative of *n* = 10 islets from 3 donors) donors (b). (c)–(e): Populational aspect of the disease progression: the onset and progression of diabetes in Ins-Fh^–/–^ mice. (c): SRB endocytotic response in Fh^–/–^ mice aged <10 weeks (early diabetes, blood glucose = 10–16 mM, *n* = 6 islets), aged >15 weeks (overt diabetes, blood glucose >30 mM, *n* = 6 islets) and wild-type littermates (*n* = 5 islets for both groups). (d): Raw images of PAPT in the islets of wild-type and Fh^–/–^ (“young” and “old”) mice, as indicated, demonstrating the heterogeneity of the response in the “young” group. (e): Distributions of the PAPT response in wild-type and Fh^–/–^ (“young” and “old”) mice. The wild-type data from both littermate groups (as in panel (c)) was pooled together.

Pancreatic islets are composed of several cell types that derive from a common lineage, possess similar K_ATP_ channel-based glucose-sensing machinery and are noted for plasma membrane excitability – but secrete different hormones that may fulfil different or even antagonistic functions.^57^ Notably, glucagon secreted by the second largest islet cellular population, α-cells, increases blood glucose when it gets dangerously low whereas insulin, in turn, is the only hormone capable of lowering blood glucose. α-Cells (identified through the targeted expression of the fluorescent reporter, Venus) displayed a mixed response to low glucose concentrations (1 mM) (Fig. 6a): there were α-cells present that had active PAPT under these conditions but around 66% of Venus-positive cells appeared inactive. The increase in extracellular glucose, as expected, suppressed secretion and PAPT in α-cells (Fig. 6a). The K_ATP_ channel inhibitor tolbutamide, which is known to produce a short-lasting stimulation of glucagon secretion, when added at intermediate or high glucose concentrations,^58^,^59^ induced a small but clear transient PAPT response in most of the Venus-positive cells (Fig. 6a). Thus, glucagon secretion in response to a physiological stimulus (low glucose) in α-cells shows great cellular heterogeneity, which brings an interesting insight into the overall mechanism of prevention of hypoglycaemia. Our data suggests that the “routine” monitoring and control of plasma glucose is performed by only a third of the α-cell population, whilst the majority of α-cells possibly serves as a strategic reserve for emergency cases and may require a stronger stimulus (such as (nor)adrenaline) to initiate secretion of glucagon. This mechanism agrees well with the earlier reports of a strong compensatory ability in the α-cell population.^60^

At the same time, δ-cells, pre-identified by RFP expressed under the somatostatin promoter,^22^ responded to glucose in a fashion similar to that of β-cells: low levels of PAPT at basal glucose, an increase at 20 mM glucose and further enhancement upon addition of tolbutamide (Fig. 6a).

These measurements in the mouse islets suggest that the endocytosis-based technology is capable of resolving secretory responses in the three major types of hormone-secreting cells in the islets. However, its clinical utility ultimately depends on its applicability to human samples. We therefore tested this in islets isolated from healthy human donors (glycated haemoglobin, HbA_1c_ <6%) and from those with type 2 diabetes mellitus (HbA_1c_ >10%). Non-diabetic islets responded with dramatically increased PAPT upon glucose stimulation, an effect that was enhanced by addition of the antidiabetic drug tolbutamide (Fig. 6b, Movie 4). However, diabetic islets did not respond to glucose and the response to tolbutamide was weak (Fig. 6b).

### PAPT provides insight into the mechanism of disease progression

We employed the endocytotic imaging technology to probe the progression of diabetes using the β-cell fumarate hydratase knock-out mouse model.^19^ These mice are born normoglycaemic but start developing a diabetic phenotype at 8–10 weeks old, which progresses to severe hyperglycaemia (fasting plasma glucose >30 mM) within a few weeks.^19^ This correlates with an age-dependent deterioration of glucose-induced insulin secretion, which is essentially normal in young/non-diabetic (<10 weeks of age) mice but severely compromised in older/diabetic mice.^19^ This progression was also detected by the polar tracer uptake (Fig. 6c). It would be natural to expect that PAPT should be progressively attenuated in the islets isolated from the mice of increasing age and indeed, in “old” mice, PAPT is strongly inhibited (Fig. 6d). However, in “young” mice, the response was attenuated only as the average value but looked very heterogeneous overall. Whereas some cells responded to glucose as strongly as wild-type cells (Fig. 6d), the responses in others were blunted in a way more similar to what was seen in the older/diabetic animals (Fig. 6d), *i.e.* displaying a bigger statistical variance (Fig. 6e). This heterogeneity would not have been detected by imaging at the whole-islet level.

## Conclusions

We conclude that the technology based on the two-photon imaging of endocytosis reliably reports the function of individual cells. It is also capable of giving insight into the disease phenotype and prevention, to a certain extent echoing such a diagnostic test as the erythrocyte sedimentation rate. Further dissecting the phenotype/genotype of the hyperglycaemia-resistant β-cells (Fh^–/–^ “young” in Fig. 6d) may provide a new avenue for tackling diseases such as type 2 diabetes mellitus. Given the pitfalls of the conventional bulk methodologies of insulin secretion (overall complexity and time intensiveness), our fairly rapid technology can be used for a quick quality assessment of the islets or other secretory tissue used for transplantation. The prolonged nature of the PAPT signal can be utilized for functional labelling of the cells, similar to Ca^2+^ labelling,^61^ for further examinations using such methods as single-cell transcriptomics.

## Abbreviations

*R*_h_: Hydrodynamic radius
PAPT: Patterned accumulation of the polar tracer
SRB: Sulforhodamine B
HbA_1c_: Glycated hemoglobin = hemoglobin fraction A_1c_
K_ATP_ channel: ATP sensitive K^+^ channel
NPY: Neuropeptide Y
MW: Molecular weight
RIA: Radioimmunoassay
ELISA: Enzyme-linked immunosorbent assay
TIRF: Total internal reflection fluorescence
SNARE: S[combining low line]oluble *N[combining low line]*-ethylmaleimide-sensitive factor a[combining low line]ttachment protein r[combining low line]e[combining low line]ceptor
FBS: Fetal bovine serum
Ins: Insulin
Glu: Glucagon
Sst: Somatostatin
RFP: Red fluorescent protein
Fh: Fumarate hydratase
ChR: Channelrhodopsin
FITC: Fluorescein isothiocyanate

## Conflicts of interest

Authors declare no conflicts of interest.

## Supplementary Material

Supplementary information

## References

[cit1] Gutierrez G. D., Gromada J., Sussel L. (2017). Front. Genet..

[cit2] Ohara-Imaizumi M., Nishiwaki C., Kikuta T., Nagai S., Nakamichi Y., Nagamatsu S. (2004). Biochem. J..

[cit3] Takahashi N. (2015). Biol. Pharm. Bull..

[cit4] Tarasov A. I., Semplici F., Ravier M. A., Bellomo E. A., Pullen T. J., Gilon P., Sekler I., Rizzuto R., Rutter G. A. (2012). PLoS One.

[cit5] Straub S. G., Shanmugam G., Sharp G. W. G. (2004). Diabetes.

[cit6] Polonsky K. S., Given B. D., Van Cauter E. (1988). J. Clin. Invest..

[cit7] Braun M., Ramracheya R., Rorsman P. (2012). Diabetes, Obes. Metab..

[cit8] Michael D. J., Ritzel R. A., Haataja L., Chow R. H. (2006). Diabetes.

[cit9] Li D., Chen S., Bellomo E. A., Tarasov A. I., Kaut C., Rutter G. A., Li W. H. (2011). Proc. Natl. Acad. Sci. U. S. A..

[cit10] Xie Z., Long J., Liu J., Chai Z., Kang X., Wang C. (2017). Front. Mol. Neurosci..

[cit11] Ryan T. A., Reuter H., Smith S. J. (1997). Nature.

[cit12] Gad H., Low P., Zotova E., Brodin L., Shupliakov O. (1998). Neuron.

[cit13] Poskanzer K. E., Marek K. W., Sweeney S. T., Davis G. W. (2003). Nature.

[cit14] Xu J., Luo F., Zhang Z., Xue L., Wu X. S., Chiang H. C., Shin W., Wu L. G. (2013). Cell Rep..

[cit15] Saheki Y., De Camilli P. (2012). Cold Spring Harbor Perspect. Biol..

[cit16] Gundelfinger E. D., Kessels M. M., Qualmann B. (2003). Nat. Rev. Mol. Cell Biol..

[cit17] MacDonald P. E., Braun M., Galvanovskis J., Rorsman P. (2006). Cell Metab..

[cit18] Wu W., Wu L. G. (2007). Proc. Natl. Acad. Sci. U. S. A..

[cit19] Adam J., Ramracheya R., Chibalina M. V., Ternette N., Hamilton A., Tarasov A. I., Zhang Q., Rebelato E., Rorsman N. J. G., Martin-Del-Rio R., Lewis A., Ozkan G., Do H. W., Spegel P., Saitoh K., Kato K., Igarashi K., Kessler B. M., Pugh C. W., Tamarit-Rodriguez J., Mulder H., Clark A., Frizzell N., Soga T., Ashcroft F. M., Silver A., Pollard P. J., Rorsman P. (2017). Cell Rep..

[cit20] Reinbothe T. M., Safi F., Axelsson A. S., Mollet I. G., Rosengren A. H. (2014). Islets.

[cit21] Reimann F., Habib A. M., Tolhurst G., Parker H. E., Rogers G. J., Gribble F. M. (2008). Cell Metab..

[cit22] Adriaenssens A. E., Svendsen B., Lam B. Y., Yeo G. S., Holst J. J., Reimann F., Gribble F. M. (2016). Diabetologia.

[cit23] Lake S. P., Bassett P. D., Larkins A., Revell J., Walczak K., Chamberlain J., Rumford G. M., London N. J., Veitch P. S., Bell P. R. (1989). Diabetes.

[cit24] Ricordi C., Lacy P. E., Finke E. H., Olack B. J., Scharp D. W. (1988). Diabetes.

[cit25] amazon.com, https://www.amazon.com/dp/B000FMWFPQ/ref=pe_385040_30332190_pe_175190_21431760_M3T1_ST1_dp_1 (accessed April 2018).

[cit26] Tarasov A. I., Semplici F., Ravier M. A., Bellomo E. A., Pullen T. J., Gilon P., Sekler I., Rizzuto R., Rutter G. A. (2012). PLoS One.

[cit27] Berg J., Hung Y. P., Yellen G. (2009). Nat. Methods.

[cit28] Panagiotidis G., Salehi A. A., Westermark P., Lundquist I. (1992). Diabetes Res. Clin. Pract..

[cit29] https://github.com/rorsmanlab/DotDetector (accessed April 2018).

[cit30] Elkin S. R., Lakoduk A. M., Schmid S. L. (2016). Wien. Med. Wochenschr..

[cit31] R Development Core Team, R: A Language and Environment for Statistical Computing, https://www.R-project.org/ (accessed April 2018).

[cit32] Takahashi N., Kishimoto T., Nemoto T., Kadowaki T., Kasai H. (2002). Science.

[cit33] Hoppa M. B., Jones E., Karanauskaite J., Ramracheya R., Braun M., Collins S. C., Zhang Q., Clark A., Eliasson L., Genoud C., Macdonald P. E., Monteith A. G., Barg S., Galvanovskis J., Rorsman P. (2012). Diabetologia.

[cit34] Low J. T., Mitchell J. M., Do O. H., Bax J., Rawlings A., Zavortink M., Morgan G., Parton R. G., Gaisano H. Y., Thorn P. (2013). Diabetologia.

[cit35] Mohammed J. S., Wang Y., Harvat T. A., Oberholzer J., Eddington D. T. (2009). Lab Chip.

[cit36] Sankar K. S., Green B. J., Crocker A. R., Verity J. E., Altamentova S. M., Rocheleau J. V. (2011). PLoS One.

[cit37] Lifson N., Lassa C. V., Dixit P. K. (1985). Am. J. Physiol..

[cit38] Nimmerjahn A., Kirchhoff F., Kerr J. N., Helmchen F. (2004). Nat. Methods.

[cit39] Aspinwall C. A., Huang L., Lakey J. R., Kennedy R. T. (1999). Anal. Chem..

[cit40] Rorsman P., Renström E. (2003). Diabetologia.

[cit41] Duvoor C., Dendi V. S., Marco A., Shekhawat N. S., Chada A., Ravilla R., Musham C. K., Mirza W., Chaudhury A. (2017). Front Physiol..

[cit42] Lougheed W. D., Fischer U., Perlman K., Albisser A. M. (1981). Diabetologia.

[cit43] Renstrom E., Ding W. G., Bokvist K., Rorsman P. (1996). Neuron.

[cit44] Armstrong J. K., Wenby R. B., Meiselman H. J., Fisher T. C. (2004). Biophys. J..

[cit45] Liu T. T., Kishimoto T., Hatakeyama H., Nemoto T., Takahashi N., Kasai H. (2005). J. Physiol..

[cit46] Oliva A., Farina J., Llabres M. (2000). J. Chromatogr. B: Biomed. Sci. Appl..

[cit47] Hvidt S. (1991). Biophys. Chem..

[cit48] Clayton E. L., Cousin M. A. (2009). J. Neurosci. Methods.

[cit49] Whim M. D. (2011). PLoS One.

[cit50] Brange J., Langkjoer L. (1993). Pharm. Biotechnol..

[cit51] Stozer A., Dolensek J., Rupnik M. S. (2013). PLoS One.

[cit52] Henquin J. C. (2009). Diabetologia.

[cit53] Gheni G., Ogura M., Iwasaki M., Yokoi N., Minami K., Nakayama Y., Harada K., Hastoy B., Wu X., Takahashi H., Kimura K., Matsubara T., Hoshikawa R., Hatano N., Sugawara K., Shibasaki T., Inagaki N., Bamba T., Mizoguchi A., Fukusaki E., Rorsman P., Seino S. (2014). Cell Rep..

[cit54] Maechler P., Wollheim C. B. (1999). Nature.

[cit55] Yoon M. S. (2016). Nutrients.

[cit56] Gooding J. R., Jensen M. V., Dai X., Wenner B. R., Lu D., Arumugam R., Ferdaoussi M., MacDonald P. E., Newgard C. B. (2015). Cell Rep..

[cit57] Rorsman P., Ashcroft F. M. (2018). Physiol. Rev..

[cit58] Cheng-Xue R., Gomez-Ruiz A., Antoine N., Noel L. A., Chae H. Y., Ravier M. A., Chimienti F., Schuit F. C., Gilon P. (2013). Diabetes.

[cit59] Kadowaki S., Taminato T., Chiba T., Nozawa M., Fujita T., Norman A. W. (1983). Endocrinology.

[cit60] Thorel F., Damond N., Chera S., Wiederkehr A., Thorens B., Meda P., Wollheim C. B., Herrera P. L. (2011). Diabetes.

[cit61] Fosque B. F., Sun Y., Dana H., Yang C. T., Ohyama T., Tadross M. R., Patel R., Zlatic M., Kim D. S., Ahrens M. B., Jayaraman V., Looger L. L., Schreiter E. R. (2015). Science.

