# Supplementary material for: Monitoring real-time hormone release kinetics via high-content 3-D imaging of compensatory endocytosis
Source: Lab Chip. 2018 Jul 30;18(18):2838–48. doi: 10.1039/c8lc00417j (PMC6250124; doi:10.1039/c8lc00417j)
Supplement: Supplementary file 1 [file LC-018-C8LC00417J-s001.pdf]

Electronic Supplementary Material (ESI) for Lab on a Chip.
This journal is © The Royal Society of Chemistry 2018

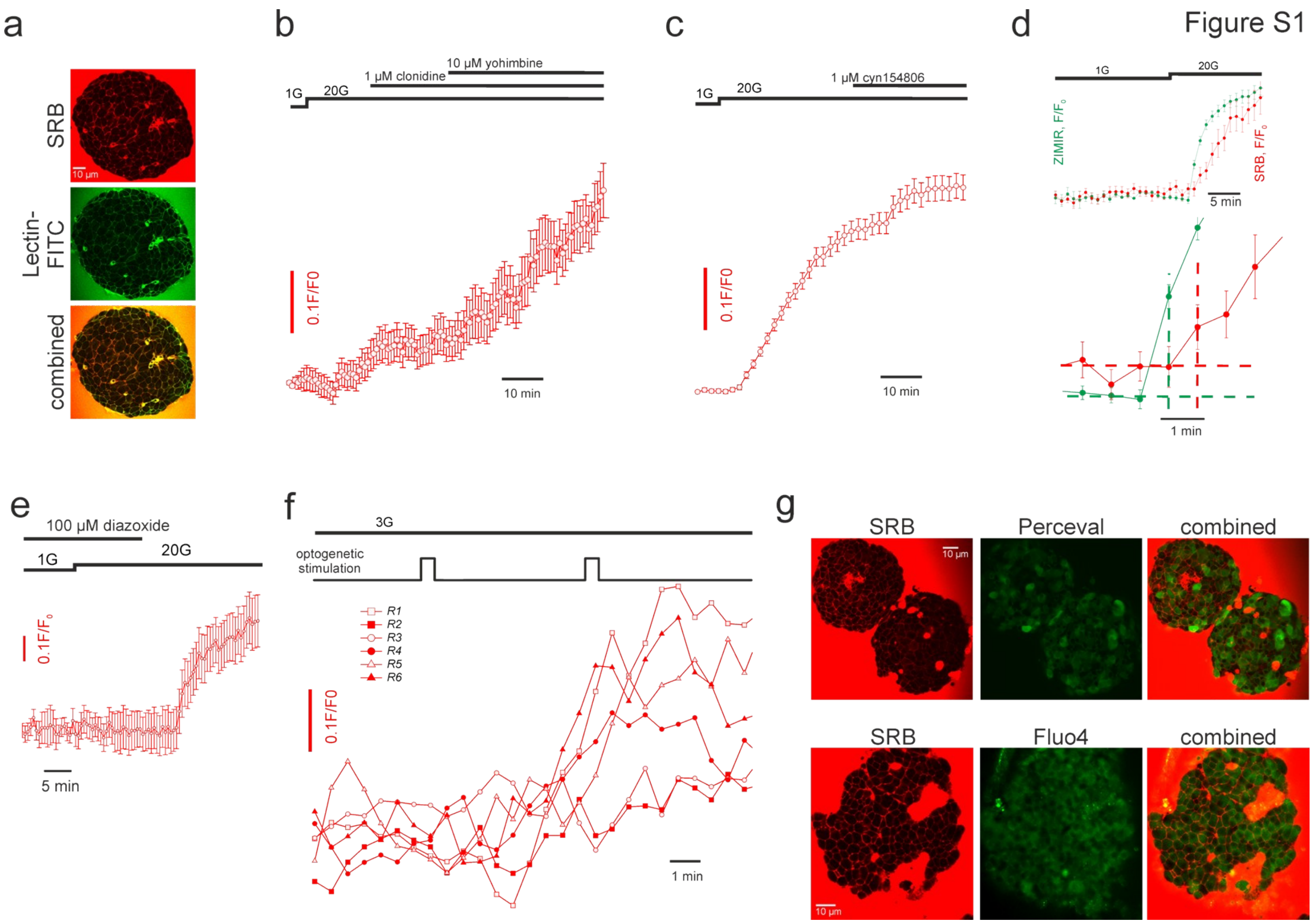

Supporting Information

Figure S1 Co-staining and co-imaging, paracrine signalling and optogenetic stimulation. (a) Co-staining of freshly isolated mouse islets with SRB and Lectin-FITC. Note the high co-localization in the vessel endothelia cells. **(b)-(c)** Effect of α_2-agonist (clonidine) and antagonist (yohimbine) **(b)** and broad range sstR-antagonist CYN154806 **(c)** on PATP. **(d)** Detailed view of the responses of the extracellular sensor Zimir and SRB patterned uptake to high glucose. **(e)** Effect of the K_{ATP} channel opener diazoxide on PAPT in pancreatic islet cells. **(f)** Kinetics of the PATP from regions *R1-R6*, as shown in Figure e. **(g)** Expression of the $[ATP/ADP]_i$ sensor Perceval (above) and trappable Ca^{2+} sensing dye Fluo4 in pancreatic islets.

Figure S2

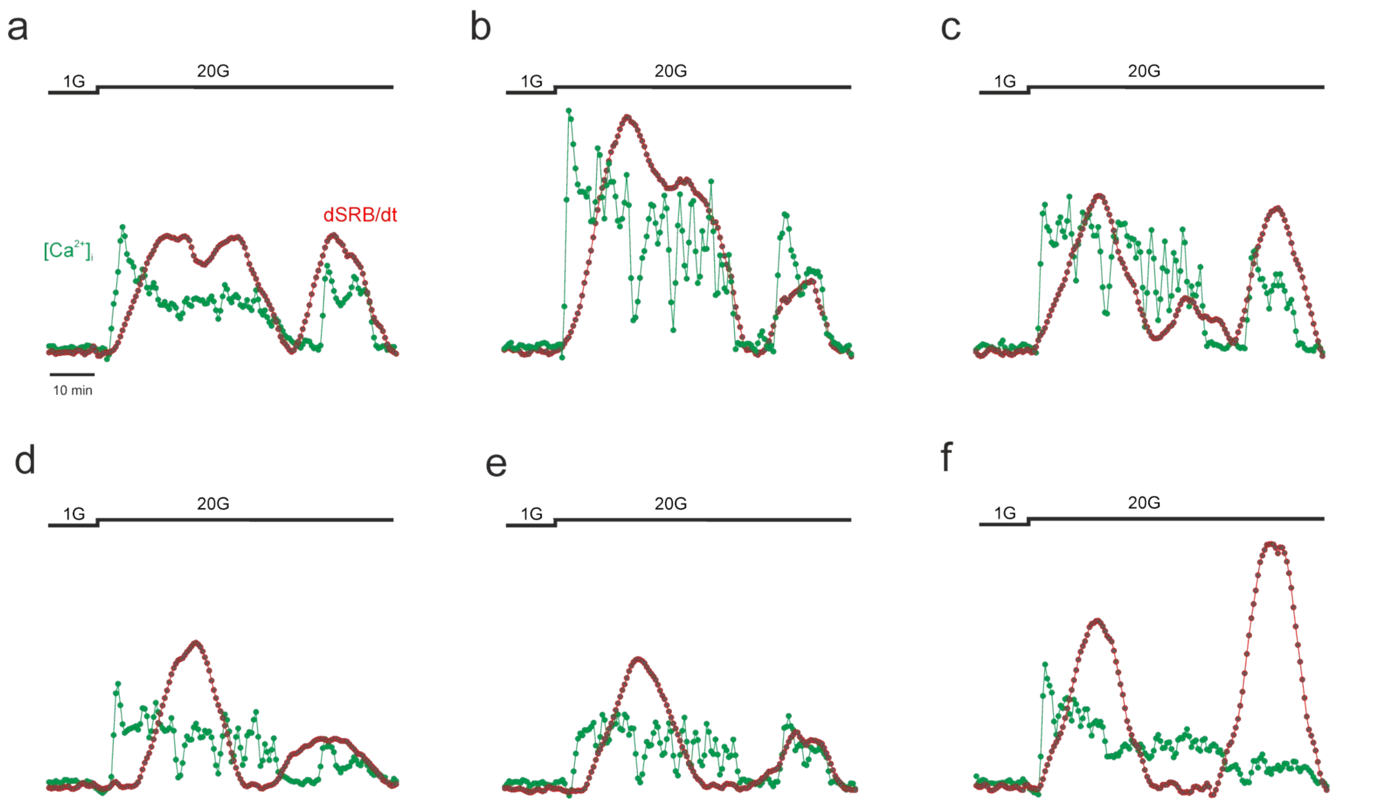

Figure S2 The coupling and the discrepancy between the $[Ca^{2+}]_i$ and PAPT response. (a)-(f): PAPT (red) and $[Ca^{2+}]_i$ response to high glucose from the same cells within an islet of Langerhans. The scale is identical for all six cells.

Movie 1 Exocytosis in pancreatic islet β-cells imaged as transient SRB Ω-shapes: https://www.dropbox.com/s/x9zrepzl7sghbho/omega_shape_exocytosis.gif?dl=0

Movie 2 Post-secretory endocytosis in pancreatic islets (2D, raw) in mouse pancreatic islets: https://www.dropbox.com/s/d8v4g3ozbnk27na/mouse_2D.gif?dl=0

Movie 3 Feature-detected 3D post-secretory endocytosis in mouse pancreatic islets: https://www.dropbox.com/s/31ufhkrpbc6j53e/3D_mouse_feature_detected.gif?dl=0

Movie 4 Post-secretory endocytosis in pancreatic islets (2D, raw) in human pancreatic islets (healthy donor):

https://www.dropbox.com/s/gdzt9bwaz1o0cuj/human_2D.gif?dl=0
